# Cognitive Predictors of Verbal Memory in a Mixed Clinical Pediatric Sample

**DOI:** 10.3390/bs3030522

**Published:** 2013-08-26

**Authors:** Lizabeth L. Jordan, Callie E. Tyner, Shelley C. Heaton

**Affiliations:** Department of Clinical & Health Psychology, University of Florida Health Science Center, PO Box 100165, Gainesville, FL 32610-0165, USA; E-Mails: callietyner@phhp.ufl.edu (C.E.T.); sheaton@phhp.ufl.edu (S.C.H.)

**Keywords:** memory, pediatric neuropsychology, cognitive development

## Abstract

Verbal memory problems, along with other cognitive difficulties, are common in children diagnosed with neurological and/or psychological disorders. Historically, these “memory problems” have been poorly characterized and often present with a heterogeneous pattern of performance across memory processes, even within a specific diagnostic group. The current study examined archival neuropsychological data from a large mixed clinical pediatric sample in order to understand whether functioning in other cognitive areas (*i.e*., verbal knowledge, attention, working memory, executive functioning) may explain some of the performance variability seen across verbal memory tasks of the Children’s Memory Scale (CMS). Multivariate analyses revealed that among the cognitive functions examined, only verbal knowledge explained a significant amount of variance in overall verbal memory performance. Further univariate analyses examining the component processes of verbal memory indicated that verbal knowledge is specifically related to encoding, but not the retention or retrieval stages. Future research is needed to replicate these findings in other clinical samples, to examine whether verbal knowledge predicts performance on other verbal memory tasks and to explore whether these findings also hold true for visual memory tasks. Successful replication of the current study findings would indicate that interventions targeting verbal encoding deficits should include efforts to improve verbal knowledge.

## 1. Introduction

Declarative verbal memory includes the processes of learning new verbal information (*i.e*., encoding words, facts, stories), storing the verbal information over time (*i.e*., retention) and, later, overtly recalling or recognizing the verbal information (*i.e*., retrieval). Research in healthy children and adults has long sought to understand the declarative verbal memory process and its underlying components. Although verbal memory problems are common in a wide variety of clinical pediatric populations (e.g., memory difficulties have been reported in children with Attention-Deficit/Hyperactivity Disorder [ADHD], Autism Spectrum Disorder [ASD], epilepsy, traumatic brain injury [TBI] and cancer), very little information is known regarding the specific verbal memory processes affected in these groups [1]. Interestingly, the nature of verbal memory problems seems to vary widely, even within specific populations (e.g., epilepsy and TBI) [2,3]. The heterogeneous nature of verbal memory problems both within and across pediatric patient populations may be related to differences in the neuroanatomical sites of disruption underlying the involved memory process. Alternatively, these verbal memory problems may be occurring secondary to impairment in other cognitive factor(s), such as poor verbal knowledge or attentional skills [4].

Research in healthy children and adolescents suggests that some memory processes occur independent from other cognitive abilities (e.g., incidental encoding and implicit recognition); however, research has revealed that the development of and normal variations in *declarative* verbal memory abilities are related to several specific cognitive domains [4,5,6]. In contrast to non-declarative memory, declarative verbal memory is considered a more “mature” form of memory, because it requires the additional act of conscious awareness when remembering verbal information. Declarative verbal memory improves with age and brain development; the maturation of the prefrontal cortex and its connections with the temporal lobe and other cortical areas contribute to declarative verbal memory [7]. Interestingly, these developmental gains in verbal memory ability also coincide with rapid development of verbal knowledge, attention, processing speed, working memory and other executive functions. Furthermore, these cognitive factors are believed to provide critical support for the maturation of declarative verbal memory strategies, which themselves become more strategic, complex and effective with age [7]. In fact, elaborative verbal encoding strategies and effortful systematic verbal retrieval searches appear to be particularly related to verbal knowledge, attention, processing speed, working memory and executive functioning [1,5,7,8,9,10]. Yet, research has not determined how difficulties in verbal knowledge, attention, processing speed, working memory and other executive functions may be related to the verbal memory problems often seen in pediatric patients with medical or developmental disorders.

Thus, the overall goal of the current study was to determine which cognitive factors contribute to delayed verbal memory performance in a large and heterogeneous sample of clinical pediatric patients. First, a factor model was developed in order to demonstrate how specific neuropsychological measures could be used to identify the discrete cognitive domains of interest in a heterogeneous pediatric sample. It was hypothesized that the cognitive model would be consistent with constructs purported by the test developers, and the model would identify factors representing verbal knowledge, attention, processing speed and working memory/executive functioning. Second, it was hypothesized that each of the cognitive factors of interest would significantly predict overall verbal memory performance. This hypothesis was based on previous literature suggesting that these cognitive domains contribute to verbal memory abilities in healthy, normally developing children. Finally, the relationships between these targeted cognitive factors and specific verbal memory component processes (*i.e*., encoding, retention and retrieval) were further explored. It was hypothesized that relevant cognitive factors would impact verbal memory performance specifically via influence on encoding and retrieval abilities.

## 2. Method

### 2.1. Participants

Data was derived from an Institutional Review Board (IRB)-approved, archival clinical study of pediatric patients who received a neuropsychological assessment at the University of Florida between 2002 and 2012. Patients were those referred for diagnostic clarification and treatment recommendations for a wide variety of childhood conditions (e.g., ASD, ADHD, learning disabilities, communication disorders), as well as cognitive disorders resulting from neurological disease or insult (e.g., TBI, stroke, epilepsy, cancer). Data for the current study was extracted for children between the ages of 5 years 0 months and 16 years 11 months who had completed the verbal memory measures of interest (*i.e*., core verbal subtests from the Children’s Memory Scale). In order to ensure that the current study results could be generalized to a broad pediatric population, patients were not excluded for any comorbid psychological or medical conditions, and there was no cut-off used to exclude patients of lower intellectual functioning.

### 2.2. Measures

Verbal memory abilities were assessed using the Children’s Memory Scale (CMS). The CMS is a comprehensive measure designed to assess learning and memory processes in children ages 5 to 16 [11]. Completion of the core battery of CMS verbal memory tests yields a norm-referenced index score of general verbal memory ability (*i.e*., Verbal Delayed Memory Index), as well as a score representing verbal encoding skills (*i.e*., Verbal Immediate Memory Index). Using recommendations from the CMS manual and communications with the test author, two additional norm-referenced verbal memory process scores were derived to represent verbal retention and retrieval skills [11,12,13]. Thus, the test scores analyzed for the current study were the CMS Verbal Delayed Memory Index (general verbal memory ability), the CMS Verbal Immediate Memory Index (encoding), a derived percent retention score (retention) and a derived delayed memory contrast score, which compared delayed recall and delayed recognition performances (retrieval).

In addition to the CMS, neuropsychological measures assessing verbal knowledge, sustained attention, working memory, processing speed and working memory/executive functioning were selected. Specifically, the following measures were used as estimates of verbal knowledge, processing speed, attention and working memory/executive functioning: from the Wechsler Intelligence Scale for Children, 4th Edition (WISC-IV), the 5 core subtests comprising the Verbal Comprehension Index (VCI) and the Processing Speed Index (PSI), as well as the Digit Span Backwards subtest [14] and from the Test of Everyday Attention for Children (TEA-Ch), the Sky Search, Score! and Creature Counting subtests [15]. Of note, the total score from the TEA-Ch Creature Counting subtest was expected to be a measure of working memory, because this score is dependent on accurate and speeded mental manipulation of numbers [15]. The other core working memory subtests of the WISC-IV (*i.e*., Digit Span Forwards, Letter-Number Sequencing) were not examined in this study in order to minimize the number of variables entered into analyses.

### 2.3. Analyses

Two multivariate statistical analyses were conducted: an Exploratory Factor Analysis (EFA) and Structural Equation Modeling (SEM). The EFA was used to construct a model where the latent factors represent cognitive domains and the measured indicators are scores from neuropsychological tests. The neuropsychological measures included in the model were the Wechsler Intelligence Scale for Children, 4th Edition (WISC-IV), core verbal comprehension subtests (*i.e*., Vocabulary, Similarities, Comprehension), the WISC-IV processing speed subtests (*i.e*., Symbol Search and Coding), WISC-IV Digit Span Backwards and three subtests of the TEA-Ch (*i.e*., Sky Search, Score!, and Creature Counting). Missing data was excluded using pairwise methodology, and an oblique rotation (Promax) was utilized to determine the factor matrices. The EFA was conducted with SPSS version 18.0.

Next, the cognitive factors identified in the EFA were entered into an SEM that investigated the relationship between the cognitive factors and a measure of general verbal memory performance (*i.e*., the CMS Delayed Verbal Memory Index score). The initial specified model was developed based on the results of the EFA. Each latent factor and any measured variables that did not load onto a factor in the EFA were predictors in the SEM. Data was examined and appeared to meet multivariate (e.g., normal distribution, homogeneity of variance, sphericity) and SEM assumptions (*i.e*., large sample size) [16,17]. All SEM analyses were conducted using the Analysis of Moment Structures (AMOS) software [18]. A special form of Maximum Likelihood (ML) estimation for incomplete data was used to obtain model fit, as well as parameter estimations, because ML is robust to missing data [17]. Models were assessed using multiple assessments of good fit (e.g., *Χ^2^*, *p* < 0.01, root mean square error of approximation (RMSEA) < 0.05) [17]. Significant parameter estimations (*i.e*., *β* weights) were identified using a *p* < 0.05 probability criterion

In addition to the multivariate analyses, a correlation matrix was used to examine the specific relationships between measures of verbal memory processes (*i.e*., CMS verbal encoding, retention and retrieval indices) and individual cognitive measures (e.g., WISC-IV Vocabulary, Comprehension and Similarities subtests), as well as overall delayed verbal memory performance. A statistical criterion of *p* ≤ 0.05, two-tailed, was used to confirm significant correlations between variables.

Finally, a mediation analysis was used to further examine the nature of the relationship between significant cognitive factors identified in the SEM and delayed verbal memory performance. Specifically, Preacher and Hayes’s bootstrapping method was used to investigate whether the relationship was mediated by the indirect effect of cognitive factors on encoding, retention and/or retrieval processes [19]. The SPSS INDIRECT macro was used to conduct the bootstrapping analysis [19]. A moderate statistical criterion (*p* < 0.05) was used to identify significant effects.

## 3. Results

### 3.1. Sample Characteristics

**Figure 1:**
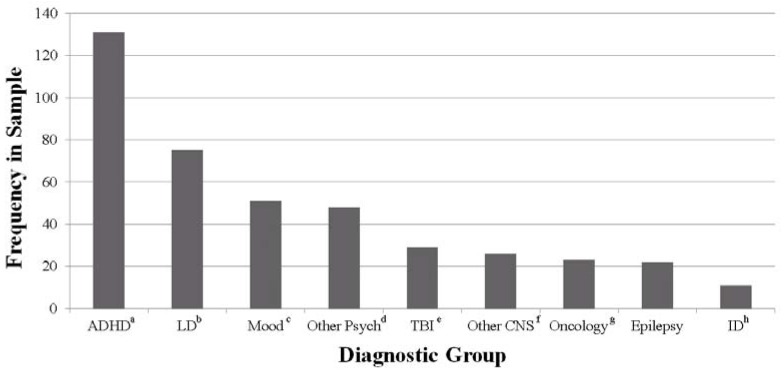
Psychiatric disorders and medical/neurological conditions represented in the study sample. ^a^ Attention-Deficit/Hyperactivity Disorder; ^b^ learning disabilities; ^c^ mood disorders (e.g., major depression, generalized anxiety disorder); ^d^ other psychiatric disorders (e.g., conduct disorder, impulse control disorder); ^e^ traumatic brain injury; ^f^ other central nervous system disorders (e.g., apraxia, sleep disorders); ^g^ oncology (e.g., brain tumor, acute lymphoblastic leukemia); ^h^ intellectual disabilities.

Review of an IRB-approved archival clinical data bank identified 571 pediatric patients who had undergone neuropsychological evaluations. Of these, 41% (*n* = 234; 139 male) had a CMS Delayed Verbal Memory Index Score and were included in subsequent analyses. The average age of the sample was 10 years old (range 5–16). The sample included 169 Caucasian (72%), 34 black (15%), nine Hispanic (4%), three biracial (1%) and one Native Hawaiian/Other Pacific Islander (< 1%) children. Additionally, 8% of the sample were of unknown race (*n* = 18). As shown in Figure 1, the sample included individuals with a variety of neurodevelopmental and acquired brain disorders, with overlap due to comorbidities reflected in the diagnostic group frequencies. Of note, over half of the sample (56%; *n* = 131) carried a diagnosis of ADHD. Furthermore, 20% (*n* = 26) of the ADHD group had a co-morbid medical disorder known to impact central nervous functioning (e.g., epilepsy, cancer, TBI). Due to the retrospective nature of this study, it was unclear what proportion of these patients with co-morbid ADHD and medical diagnoses had ADHD symptoms that were directly related to the medical disorder (e.g., ADHD symptoms onset secondary to TBI) *versus* symptoms that were clearly unrelated to the medical diagnosis (e.g., documented ADHD symptoms pre-dating TBI). Furthermore, though diagnostic criteria fail to provide explicit guidelines regarding comorbid diagnosis of ADHD in such situations, we acknowledge that there is considerable variability in the diagnostic practices of pediatric neuropsychologists. Thus, it is worth noting that the current sample is known to include children who met diagnostic criteria for ADHD [20], but underlying etiology for each case is unknown.

Descriptive information about the sample’s cognitive and verbal memory abilities are presented in Table 1. Although the sample included patients with a wide range of functioning, the overall sample demonstrated normal intelligence (WISC-IV Full Scale Intelligence Quotient = 90.40, *standard deviation* = 14.85, range = 40−130), verbal knowledge, processing speed, attention, working memory/executive functioning and delayed verbal memory abilities. Of note, only 14 of the patients (6%) had a WISC-IV Full Scale Intelligence Quotient lower than 70. 

**Table 1:** Cognitive and verbal memory test performance of sample.

Cognitive DomainTest Variable	Score Type	Mean	SD	n
**Verbal Knowledge**				
Vocabulary (WISC-IV)	ss	8.47	3.07	195
Similarities (WISC-IV)	ss	9.28	3.24	195
Comprehension (WISC-IV)	ss	9.32	3.04	195
**Processing Speed**				
Symbol Search (WISC-IV)	ss	8.13	3.14	200
Coding (WISC-IV)	ss	7.32	3.32	201
**Attention**				
Sky Search—Attention (TEA-Ch)	ss	7.97	3.09	204
Score! (TEA-Ch)	ss	7.35	3.60	209
Creature Counting—Total (TEA-Ch)	ss	8.13	3.57	179
**Working Memory**				
Digit Span Backward (WISC-IV)	ss	7.73	3.01	136
**Verbal Memory**				
Delayed Recall Score (CMS)	StS	92.58	17.42	233

Notes: WISC-IV = Wechsler Intelligence Scale for Children, 4th Edition; TEA-Ch = Test of Everyday Attention for Children; CMS = Children’s Memory Scale; ss = scaled score (*m* = 10, *sd* = 3); StS = standard score (*m* = 100, *sd* = 15).

### 3.2. Exploratory Factor Analysis (EFA)

An EFA was conducted on the nine cognitive measures. Multivariate assumptions of no multicollinearity, singularity and sphericity were met [16]. Based on an *a priori* hypothesis that four factors would be identified, an initial analysis was run to obtain eigenvalues for a four-factor EFA. Three components had eigenvalues over Kaiser’s criterion of one, which, in combination, explained 63% of the variance. The fourth identified factor did not meet Kaiser’s criterion; however, the final sample included in the EFA (*n* = 196) was somewhat small, and only one observed measure (Digit Span Backwards) loaded onto this factor. In addition, the fourth factor meets Joliffe’s criterion (eigenvalue > 0.7), so it was retained in the final analysis. Table 2, Table 3 show the pattern and structure matrix of the factor loadings after oblique rotation. Although cross-loadings were present in the pattern matrix, the measures that clustered on the same factors suggest that Factor 1 represents verbal knowledge, Factor 2 represents processing speed, Factor 3 represents attention and Factor 4 represents working memory.

**Table 2:** Pattern matrix of rotated factor loadings from exploratory factor analysis.

Measure	Factor 1	Factor 2	Factor 3	Factor 4
	Verbal Knowledge	Processing Speed	Attention	Working Memory
Vocabulary (WISC-IV)	0.92 *			
Similarities (WISC-IV)	0.93 *			−0.13
Comprehension (WISC-IV)	0.76 *	0.13		0.14
Symbol Search (WISC-IV)	0.11	0.78 *		0.15
Coding (WISC-IV)		0.88 *		
Sky Search—Attention (TEA-Ch)		0.43	0.59 *	−0.14
Score! (TEA-Ch)		0.22	0.55 *	
Creature Counting—Total (TEA-Ch)		−0.33	0.81 *	0.16
Digit Span Backward (WISC-IV)		0.10		0.97 *

Notes: the extraction method was Principal Component Analysis; the rotation method was Promax with Kaiser Normalization; rotation converged in six iterations; * measures that loaded well on a common factor as indicated by the pattern and structure matrices; WISC-IV = Wechsler Intelligence Scale for Children, 4th Edition; TEA-Ch = Test of Everyday Attention for Children.

**Table 3:** Structure matrix of rotated factor loadings from exploratory factor analysis.

Measure	Factor 1	Factor 2	Factor 3	Factor 4
	Verbal Knowledge	Processing Speed	Attention	Working Memory
Vocabulary (WISC-IV)	0.91 *	0.16	0.29	0.27
Similarities (WISC-IV)	0.89 *	0.16	0.25	0.16
Comprehension (WISC-IV)	0.83 *	0.30	0.27	0.40
Symbol Search (WISC-IV)	0.32	0.82 *	0.26	0.27
Coding (WISC-IV)	0.14	0.86 *	0.15	
Sky Search—Attention (TEA-Ch)	0.12	0.53	0.63 *	
Score! (TEA-Ch)	0.21	0.34	0.59 *	
Creature Counting—Total (TEA-Ch)	0.28	−0.12	0.79 *	0.31
Digit Span Backward (WISC-IV)	0.28	0.20	0.21	0.97 *

Notes: the extraction method was Principal Component Analysis; the rotation method was Promax with Kaiser Normalization; rotation converged in six iterations; * measures that loaded well on a common factor as indicated by the pattern and structure matrices; WISC-IV = Wechsler Intelligence Scale for Children, 4th Edition; TEA-Ch = Test of Everyday Attention for Children.

### 3.3. Structural Equation Model (SEM)

**Figure 2:**
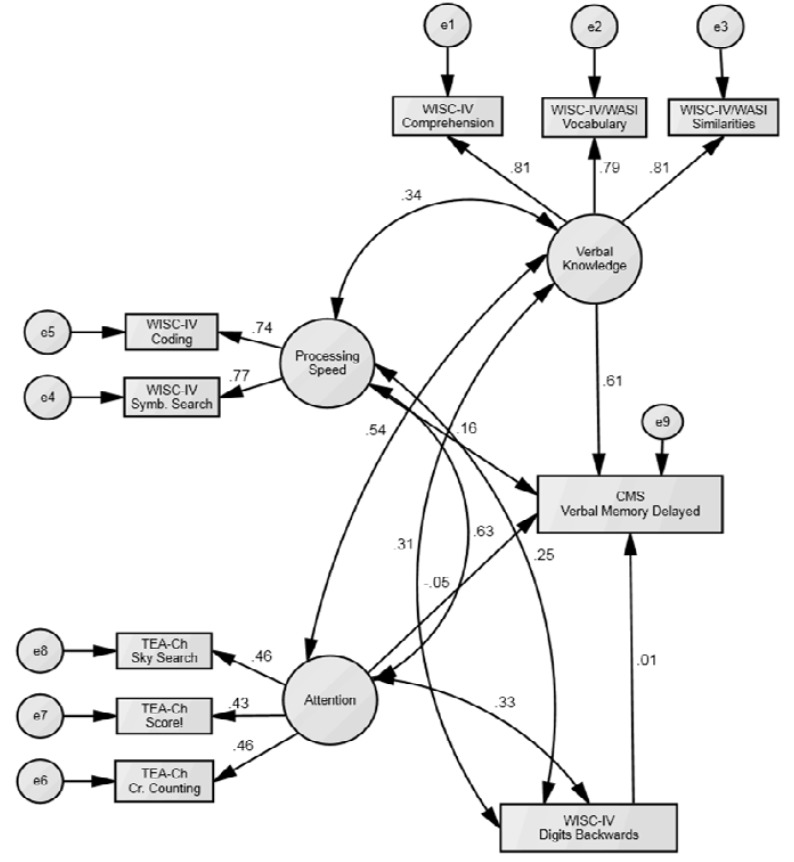
Final structural equation model (SEM) of factors contributing to verbal memory performance. The figure shows the final SEM with the standardized regression estimates added. Circles represent latent variables (*i.e*., the cognitive factors), and rectangles represent measured variables. Double-headed arrows indicate covariance, whereas single-headed arrows represent one-way relationships. (WASI)

An SEM was developed based on the previous EFA and was tested using data from the sample of 234 pediatric patients (See Figure 2 for an illustration of the SEM model). Multiple goodness-of-fit test statistics, which are displayed in Table 4, indicated that the model was satisfactory [21]. Consistent with the EFA, the SEM revealed significant relationships between factors and their corresponding variables (*p* < 0.01). Within each factor, the standardized regression estimates were comparable. Finally, the SEM revealed that only the Verbal Knowledge factor predicted delayed verbal memory performance (*β* = 0.61, *p* < 0.01). Factors representing processing speed and attention, as well as the measure of working memory did not explain a significant amount of variance within a measure of delayed verbal memory*.*

**Table 4:** Goodness-of-fit statistics for the final structural equation model of factors contributing to verbal memory performance.

Model	*Χ^2^*	CMIN/DF	NFI	RFI	RMSEA	RMSEA sig.
Independence (Null) Model 1	-	10.41	0.00	0.00	0.20	0.00
Specified Model 1	73.78 **	1.99 **	0.87	0.81	0.06	0.11

Notes: * *p* < 0.05; ** *p* < 0.01; CMIN/DF = minimum discrepancy divided by degrees of freedom; NFI = normed fit index; RFI = relative fit index; RMSEA = root mean square error of approximation; RMSEA sig. = RMSEA significance; AIC = Akaike Information Criterion; a model with “good fit” is indicated by the following measures: CMIN/DF, NFI and/or RFI are close to 1.00, RMSEA < 0.10 and non-significant.

### 3.4. Correlation and Bootstrapping Analyses to Examine Possible Mediation

The correlation matrix revealed significant relationships between the measure of overall delayed verbal memory performance and individual measures of verbal knowledge, verbal encoding and verbal retrieval abilities (Table 5).

**Table 5:** Correlations between discrete verbal memory processes, overall verbal memory performance and measures of verbal knowledge.

Verbal Memory Process	Verbal Delayed Index (CMS)		Vocabulary (WISC-IV)		Similarities (WISC-IV)		Comprehension (WISC-IV)	
*CMS Score*	*r*	*n*	*r*	*n*	*r*	*n*	*r*	*n*
Encoding *Immediate Memory*	0.77 **	232	0.51 **	193	0.49 **	193	0.51 **	193
Retention *Retention*	0.04	180	−0.03	150	0.10	150	−0.01	150
Retrieval *Delayed Memory Contrast*	0.44 **	212	0.06	176	0.15 *	176	0.08	176

Notes: *r* = Pearson correlation; retention and retrieval measures were derived from the CMS’s normative sample [11,12,13]; * *p* < 0.05; ** *p* < 0.01; CMS = Children’s Memory Scale; WISC-IV = Wechsler Intelligence Scale for Children, 4th Edition.

**Figure 3:**
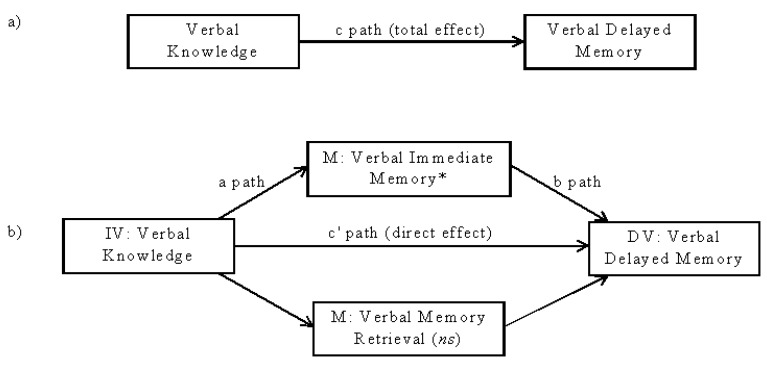
Specified model for bootstrapped mediation analyses. (a) Total effect of verbal knowledge on verbal delayed memory; (b) mediation model testing two possible mediators: verbal immediate memory (encoding) and verbal memory retrieval; * *p* < 0.05; *ns* = non-significant.; IV = independent variable; DV = dependent variable; M = mediator.

Mediation analyses using bootstrapping methodology further revealed that the significant relationship between verbal knowledge and delayed verbal memory performance was significantly and partially mediated by the effect of verbal knowledge on verbal encoding abilities. See Figure 3 and Table 6 for the bootstrapped mediation model and results. The effect of verbal knowledge on verbal retrieval abilities was not a significant mediator (*p* > 0.05).

**Table 6:** Total and direct effect of verbal knowledge on verbal delayed memory, and the total indirect effect of verbal knowledge on verbal delayed memory via verbal immediate memory.

Dependent variable	Total effect of IV on DV (path c)	Direct effect of IV on DV (path c’)	Total indirect effect of IV on DV via MV (path ab)
	*B* (*SE*)	*B* (*SE*)	*B* (*SE*)
Delayed Memory *CMS Verbal Delayed Memory Index Score*	0.73 * (0.08)	0.20 * (0.06)	0.53 * (0.07)

Notes: * *p* < 0.001; *B* = un-standardized coefficient; *SE* = standard error; IV = independent variable (*i.e*., verbal knowledge as measured by WISC-IV VCI); DV = dependent variable (*i.e*., CMS Verbal Delayed Memory Index Score); MV = mediator variable (*i.e*., CMS Verbal Immediate Memory Index Score); CMS = Children’s Memory Scale; WISC-IV = Wechsler Intelligence Scale for Children, 4th Edition; VCI = Verbal Comprehension Index.

## 4. Discussion

### 4.1. Verbal Knowledge Significantly Contributes to Verbal Memory Performance

This study identified a model of cognitive factors contributing to delayed verbal memory performance in a heterogeneous clinical pediatric sample, with a significant relationship noted between verbal knowledge and delayed verbal memory performance. The identified model used neuropsychological measures to represent four distinct cognitive domains. Overall, the model was consistent with the cognitive constructs purported by the test developers [15,22,23]. Furthermore, the model replicated previous studies reporting this relationship in normally developing individuals [5,7,8,10,11]. Importantly, this is the first known study to report that verbal knowledge significantly predicted delayed verbal memory performance in a large, heterogeneous *clinical* pediatric sample.

### 4.2. The Relationship between Verbal Knowledge and Verbal Memory Performance is Mediated by Verbal Encoding Abilities

Previous researchers have proposed that the cause-and-effect relationship between verbal knowledge and verbal memory is bi-directional, such that memory influences knowledge and, at the same time, knowledge impacts memory [5]. However, it can also be argued that the relationship between verbal knowledge and verbal memory is *primarily* uni-directional, such that increased knowledge directly improves verbal memory abilities [5,7,8,10]. Evidence for this uni-directional relationship has been historically described in both cognitive development and behavioral psychology. For example, healthy, young children can display verbal knowledge (e.g., through use of oral language and verbal behavior) as early as three years old; however, healthy children do not clearly demonstrate declarative verbal memory abilities until later in age [24]. Use of verbal encoding strategies emerges between the ages of seven to 10; in normal development, older children demonstrate more organized, semantic-based clustering of verbal information during learning trials [1,5,7]. Dehn theorized that verbal knowledge provides structure for appropriate understanding, rich elaboration and formation of associations between old and newly learned verbal information [1]. Thus, verbal knowledge may primarily affect verbal memory through its influence on verbal *encoding* processes. Consistent with the existing literature, the results of this study’s mediation analysis provides empirical evidence that verbal knowledge influences verbal memory performance by way of its influence on the verbal encoding process.

### 4.3. Other Cognitive Factors Do Not Significantly Contribute to Verbal Memory Performance

Previous literature has reported that processing speed, attention and working memory/executive functioning abilities influence verbal memory performance in healthy children and adults [1,6,7,11,25,26,27,28]. However, the current SEM results failed to identify a significant relationship between these cognitive factors and verbal memory performance. These null findings were not explained by problems with multicollinearity. Although previous research has described the relationship between verbal memory performance and *individual* cognitive domains (e.g., processing speed and memory in TBI) [29], this is the first known study to examine the *simultaneous* relationships between multiple cognitive domains and verbal memory of a clinical pediatric sample. In other words, this is the first known study to examine the *relative* amount of variance in memory explained by separate cognitive domains. The SEM results demonstrate that when verbal knowledge is included in a model of verbal memory, the remaining cognitive factors did not explain a significant amount of additional variance in memory performance.

### 4.4. Study Limitations

The major limitations of this study are related to the nature of the study sample. This study examined data from patients who were able to complete the CMS verbal memory subtests. The review of archival clinical data identified 571 pediatric patients who had undergone neuropsychological evaluations. Of these, less than half (*n* = 234) of the pediatric patients met inclusion criteria for analysis, because the majority of patients were missing the required CMS memory measure. Due to the retrospective nature of this study, it is unclear why so many of the patients initially identified in the archival clinical data review were missing CMS scores. It is possible that a portion of the excluded patients were outside of the CMS normative age range of five years zero months and 16 years 11 months. However, the study’s inclusion criteria may have introduced sample bias, because patients with very low intelligence and/or insufficient oral language may not have been able to complete the CMS. Thus, generalizability of the study findings may be limited. Future studies should aim to replicate this study’s findings through prospective research in clearly representative samples. Alternatively, different verbal memory measures that accommodate a wider age range and range of functioning could be used.

In addition, this study sample was heterogeneous. Based on the variety of medical and psychological diagnoses present in the sample, as well as the wide range of cognitive abilities, the sample likely includes individuals at different levels of neuropsychological development and with various severities of neurological compromise. Though the sample composition was purposefully kept broad in order to maximize the generalizability of our findings, it is possible that the heterogeneous nature of our sample produced large variability that reduced the ability of the statistical analyses to detect significant relationships between verbal memory and other cognitive domains. Future research should determine whether the multivariate relationships between cognitive domains and verbal memory vary between specific clinical pediatric subgroups.

## 5. Conclusions

The goal of this study was to extend previous research conducted in healthy adults and children to a clinical pediatric sample. Pediatric patients are often referred for neuropsychological evaluation and treatment of verbal “memory problems”; however, previous research has not described the exact nature of these difficulties within a memory process model, nor which cognitive factors might contribute to identified memory problems. This study found that in a large, heterogeneous sample of pediatric patients, verbal knowledge skills are positively associated with performance on verbal memory tasks. No other measured cognitive factors were significantly related to verbal memory performance. This finding was consistent with existing literature that reports that verbal knowledge is related to verbal memory abilities in healthy, normally developing children. The exact causal nature of this relationship remains unclear; however, these current results support the theory that improving verbal knowledge will improve verbal encoding, which, in turn, will positively impact overall verbal memory skills. This hypothesis may have important treatment implications. Specifically, this raises the possibility of improving verbal encoding abilities (and, thus, verbal memory skills) by directing cognitive rehabilitation efforts toward improved verbal knowledge. If the current study findings are replicated, we would recommend future rehabilitation studies that seek to identify methods for improving verbal knowledge in pediatric patients with memory difficulties and investigate whether interventions targeting verbal knowledge skills result in additional verbal memory encoding (and associated verbal memory) improvements.
